# Supplementary figures and images for: Deep autoencoder-based behavioral pattern recognition outperforms standard statistical methods in high-dimensional zebrafish studies
Source: PLoS Comput Biol. 2024 Sep 10;20(9):e1012423. doi: 10.1371/journal.pcbi.1012423 (PMC11414989; doi:10.1371/journal.pcbi.1012423)

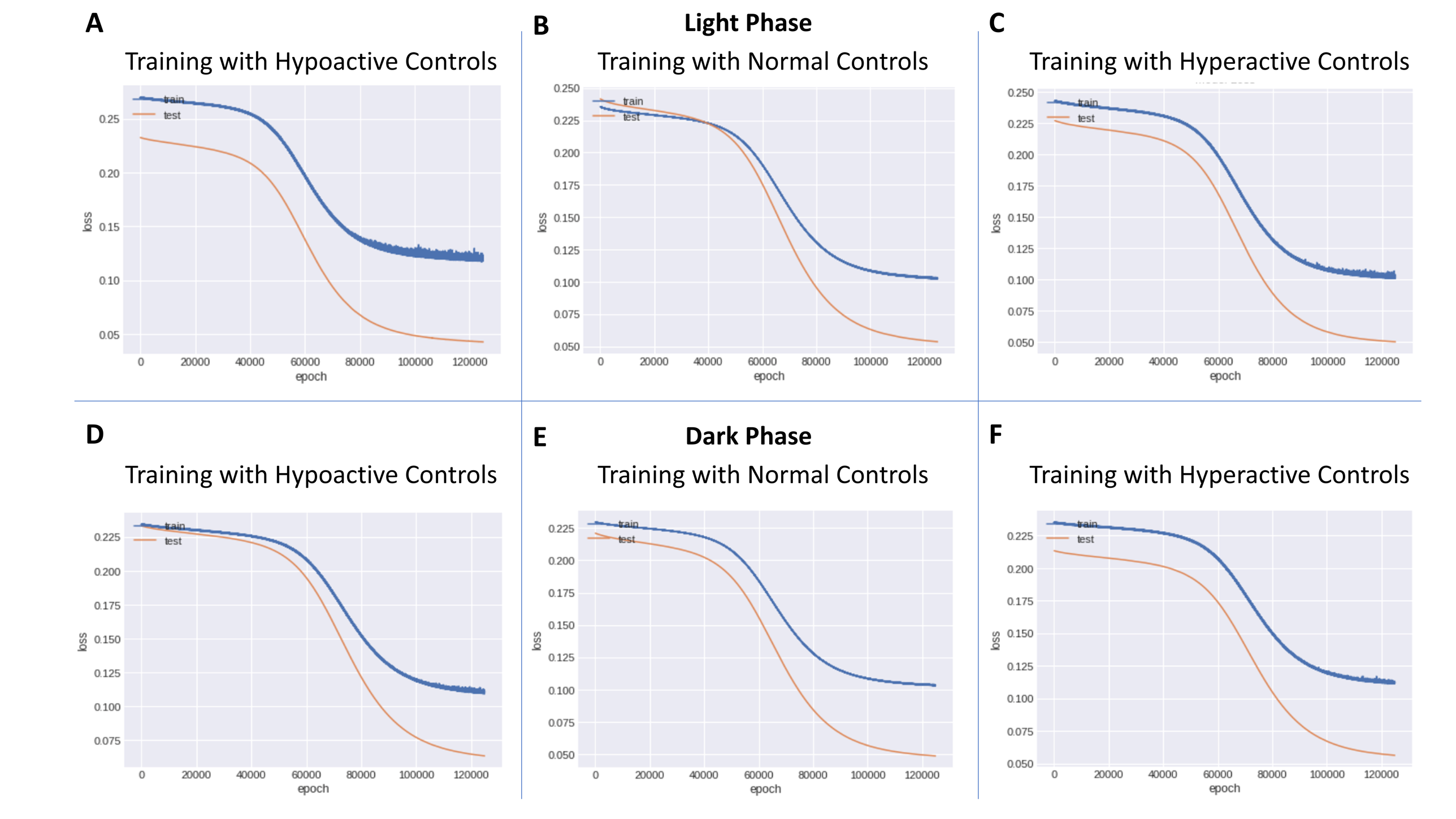

Supplement: S1 Fig — Changes of loss functions during the training of (A) light-hypoactive controls, (B) light-normal controls, (C) light-hyperactive controls, (D) dark-hypoactive controls, (E) dark-normal controls, (F) dark-hyperactive controls. Blue line–training data (controls-only), orange line–test data (abnormal-only). (TIF) [file pcbi.1012423.s003.tif]

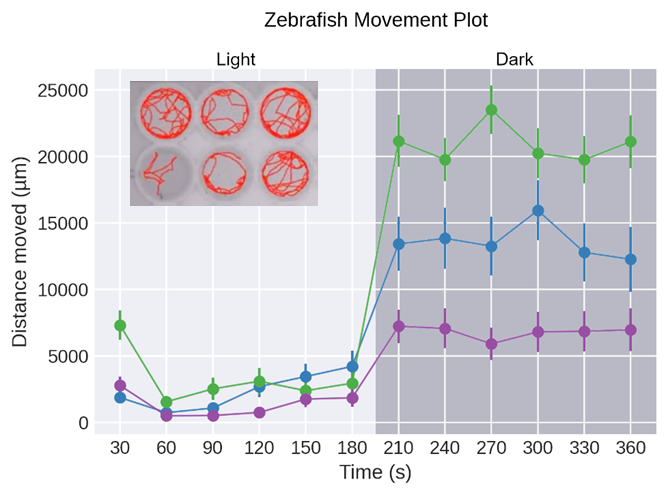

Supplement: S2 Fig — Zebrafish larvae were statically exposed to a chemical from six hpf until five dpf. At five dpf, behavior was measured under environmental conditions of continuous light for three minutes (0–180) followed by three minutes of dark (180–360). This plot shows representative control behavior data (n = 7 per line) classified as hyperactive (blue line), normal (green line) or hypoactive (purple line). The insert shows an example of larval behavioral tracks produced by Ethovision XT software. Figure depicts means ± SEM. (TIF) [file pcbi.1012423.s004.tif]
