# Supplementary material for: Deep autoencoder-based behavioral pattern recognition outperforms standard statistical methods in high-dimensional zebrafish studies
Source: PLoS Comput Biol. 2024 Sep 10;20(9):e1012423. doi: 10.1371/journal.pcbi.1012423 (PMC11414989; doi:10.1371/journal.pcbi.1012423)
Supplement: Attachment — Submitted filename: PCOMPBIOL-D-23-00581_R1_Author response to Reviewers.docx [file pcbi.1012423.s005.docx]

Reviewer's Responses to Questions

**Comments to the Authors:
Please note here if the review is uploaded as an attachment.**

Reviewer #1: Overall the authors did a could job at highlighting why one would want to opt to use deep autoencoders when analyzing behavioral data. A few sections were hard to follow. For example, the authors refer "traditional statistical methods" throughout the manuscript and vaguely comment on some of the caveats of the such methods for analyzing toxicological behavioral data. But the latter is done only at a superficial level. The authors don't put forward enough the intricacies of the toxicological behavioral data. What is particular about those data? Moreover, what are the specific caveats of the traditional statistical methods? Is one of the main findings of this study that autoencoders helped to discriminate between normal and abnormal behaviors for even very low concentration of toxins?

Thank you so much for your thoughtful and constructive feedback on our manuscript. We greatly appreciate the time and effort you invested in reviewing our work. In response to your comments, we have made the following major changes:

- Provided additional statistical analysis details to both the results and methodology sections.

Below I provide further details of the aforementioned sections that could be rephrase to both improve flow and clarity of the manuscript -- especially for readers who might not be familiar to such data or methods.

L19: I would suggest to change to "model organism" rather than tool.

Thank you, we’ve updated the language in the text.

L75: How is this finding specifically crucial for public health? Be more specific

Thank you for this comment. We have added the following statement to the Author summary: “this is a crucial finding for the protection of public health as exposure can lead to a range of neurodevelopmental disorders, including cognitive deficits and behavioral problems.”

L81-82: "...making it applicable to multiple laboratories" Do the authors mean that due the high flexibility of autoencoders, other people working on similar type of highly variable data would be able to apply similar network architecture to their data?

We agree that this is vague and added the following clarification to the Author Summary: “thereby making it applicable to multiple laboratories generating similar data.”

L138: what does "dpf" stands for?

This stands for days post-fertilization (dpf) and can be found on line 117 of the revised text.

L138-142: It would be great if the authors could be more specific by elaborating how anomaly detection could potentially solve that issue mentioned in the example rather than vaguely mentioning that this issue could be tackled by machine learning. Perhaps the authors can specify the exact issues, from an analytical point of view, how does the traditional methods not account for them? How does anomaly detection provide an alternative path to solve this issue?
L158: "traditional statistical methodologies" - this is very vague, which methods are the authors specifically referring to?

L231: Do the authors refer to K-S test as the traditional statistical analysis?

We agree and have changed the Introduction section test: “The performance of our deep autoencoders was compared against a two sample Kolmogorov–Smirnov test (K-S test), a standard for behavioral assessment.”

L354 -- L363: In general I had a hard time understanding how the K-S test were performed? Did the authors compare the empirical cumulative distributions of individuals from different groups (hypo-, normal, hyperactive) to their controls to classify whether they were normal vs abnormal? What is the threshold of "normal" v/s "abnormal" behaviors?

Thank you for this comment. We have updated the Methods sections as follows: “A two sample Kolmogorov–Smirnov test (K-S test), a non-parametric two-sided test with no adjustments for normality or multiple comparisons, was used to compare each chemical-concentration combination with their respective same plate controls (p < 0.05).”

Reviewer #2: The authors present a timely study concept of the implementation of autoencoder-based pattern recognition that is described as an improvement upon standard statistical methods of classification of zebrafish exposed to various neurotoxic chemical treatments. Despite a useful discussion of the need for more sophisticated analysis methods to be applied to behavioral classification as these techniques are increasingly implemented in various fields, the authors have not established a correct standard in the field for the comparison base that they use for their main analysis design.

Thank you so much for this helpful feedback. We appreciate your thoughtful comments and insights to enhance the quality of our manuscript. In response to your comments, we have made the following major changes:

- We have calculated and assessed angular velocity using a two sample Kolmogorov–Smirnov test in a similar manner to our evaluation of distance moved. The details have been added to the Methods and Results sections.

A major drawback of the design of the study is that the authors frame all improvements with their autoencoder method against basic statistical methods that are applied to classification based upon a metric of distance traveled, velocity, x-position, and y-position. Since each of these 4 measures are discussed, it is unclear to the reader whether all 4 of these measures were used for the statistical model or if only distance traveled is used. Please clarify this in the revision.

We agree that this is an important clarification and have added the following text to the results section: “A two sample Kolmogorov–Smirnov test (K-S test) was used to compared treated vs control distance moved and angular velocity in light/dark cycling in zebrafish larvae at five dpf.”

This measure of distance traveled is not the typical standard in the field for classification of subjects that are treated with toxins. Rather, distance traveled is often utilized as a locomotor control, which often is not capable of detecting variation between subjects treated with a toxin.

If locomotor impairment in the form of reduced distance traveled is found, this standardly indicates that subjects are not able to demonstrate more complex effects of toxin exposure. Rather reduced distance traveled would show that subjects display gross locomotor impairment, which could be attributed to multiple effects. This gross locomotor impairment could be due to inhibition of motor control or behavioral impacts and cannot be attributed to either effect as they are confounded with one another when dose of toxin is high enough to induce locomotor impairment. For the reasons discussed above, other metrics of behavioral consequences of toxin exposure including angular velocity and turn angle are utilized by many of toxicological studies utilizing the zebrafish model as they are sensitive to toxin effects, which may otherwise not be measured using metrics of distance traveled and velocity alone.

This standard of comparison of the autoencoder method against the distance traveled / velocity metric is flawed for these reasons. As such, it would be a great improvement upon the basic points of this study if a more standard metric such as angular velocity, or turn angle is used as the comparison base for classification using standard statistical methods. Otherwise, it is already expected that the distance traveled metric should not vary across subjects if the subjects are receiving a dose of toxin that is known to cause a behavioral impact without gross locomotor impairment (since distance traveled is a locomotor control and is not suggested as a metric for differentiating subjects using standard methods).

The basic point of the deep autoencoder improving upon standard methods would be greatly strengthened if the authors perform an analysis based on metrics that are more established for assessing subtle toxic effects (that are shown even when distance traveled does not change, such as changed angular velocity or changed turn angle, compared to controls.) Please address some form of an analysis of metrics other than distance traveled and velocity as the basis of comparison against the autoencoder-based strategy this in revision of this manuscript.
Is there any improvement of the autoencoder strategy compared to metrics such as angular velocity and turn angle? If there is no additional improvement from the autoencoder strategy when using these more typical measurements of subtle toxic effects, then the authors should declare this in the manuscript for full transparency and for generalizability of their findings to the field as a whole, in which these measures are commonly utilized.

In response, we carried out additional analyses using angular velocity. We calculated and assessed angular velocity using a two sample Kolmogorov–Smirnov test in a similar manner to our evaluation of distance moved. These results have been added to the Statistical classification of behavior section: “We identified 39 chemical-concentration combinations from ten chemicals and 26 chemical-concentration combinations from 10 chemicals capable of inducing a significantly different (p < 0.05) behavioral response using both distance moved (Supp. Table 2) and angular velocity (Supp. Table 3), respectively. While 10 chemicals were identified using both methods, nine chemicals were similar, with distance moved finding a significant difference in 2-methylphenanthrene at 100 µM and angular velocity finding a difference in sodium 2-(N-ethylperfluorooctane-1-sulfonamido)ethyl phosphate at 0.25 µM. Considering that distance moved revealed more chemical-concentration combinations in this screening application, we used this metric to identify abnormal larvae to ensure a sufficient number for training the autoencoder models.”

Further, we emphasize here that in this early-life-stage, high-throughput application, metrics that we might consider in juvenile or adult fish (turn angle, etc.) for characterizing specific behaviors were not the primary aim.

I have discussed this point in detail since it is essential to address to improve the theoretical basis of the study design. Aside from this point, there are some additional points below. It is uncertain how the doses of toxins were chosen in this study. Were they based on the literature from other researchers or arrived at due to preliminary / unpublished studies within the authors’ lab or previously published work within the authors' lab. In either case, it should be specifically cited how these doses were arrived upon and whether the authors assessed standard metrics for establishing dose such as LD50 for each toxin. Please address this in revision of this manuscript.

Thank you for bringing this oversight to our attention. The following details have been added to the Developmental chemical exposure section: “The concentrations evaluated were based on preliminary studies within the authors’ lab to span lethal and sub-lethal concentration range were possible based on physical chemicals properties including solubility.”

It is unclear to the reader how the authors established the subjects as hyperactive, normal, or hypoactive. Was this based on categorical splitting of the data into equally numbered groups as is done with a traditional median split? Or were these classes based on specific cut-off points of the subjects based on other studies in the field which have standard cut-offs for these metrics? Please address this in revision of this manuscript.

Thank you for this comment. As suggested, we have added additional details to the Methods - Statistical analysis section: “Interexperimental zebrafish larval response to light/dark cycling is highly variable (Supp Fig 2). Therefore, it was essential to group the unexposed controls based on the mean from individual 96-well plates compared to mean movement for unexposed controls across all plates. Controls from individual plates with statistically significant (p < 0.01) differences in movement compared to the average of all controls were grouped together as hyperactive, normal, or hypoactive. Following grouping the K-S test was used to compare each chemical-concentration combination with their respective same plate control (p < 0.05). Individuals in the 30th and 70th percentiles of each chemical-concentration combination were defined as abnormal.”

The discussion section would benefit from some explanation of the purported reasons why certain classes of chemicals are better differentiated by the autoencoder-based method vs. standard statistical methods. Does this vary according to chemical class / structure?

Thank you for this excellent question. It does appear that the autoencoder is better than distance moved and angular velocity in identifying PFAS induced behavioral alternations. We have added the following text to the Discussion section: “While making a definitive claim will require further experimentation, it does appear that the autoencoder method is particularly sensitive at detecting changes due to PFAS exposure. PFAS are associated with increased glutamate levels in the hippocampus and catecholamine levels in the hypothalamus, decreased dopamine in the whole brain after PFAS exposure, and increased extracellular glutamate has been observed in the hippocampus epileptic rats (Brown-Leung & Cannon, 2022; Soukupova et al., 2015). Thus, it is reasonable to infer that these neurochemical changes are capable of altering autoencoder-detectable patterns without changing locomotor magnitude or direction.”

It is unclear what type of analysis was performed in Figure 4 to determine that the trained model and the test dataset’s model produce similar results or if this is simply a qualitative observation that the training and test data models seems similar to the authors. It would be valid to claim similar results on the basis of a non-significant statistical test, rather than just a visual inspection.

We agree that a statistical test is required rather than visual inspection and details of the analysis have been included in the Methods - Experimental confirmation of autoencoder findings section: “Following model development two chemicals were identified for follow-up laboratory testing. We generated new data using 2-Methylphenanthrene, and Nonafluoropentanamide. 2-Methylphenanthrene was chosen as the autoencoder identified it was different from controls at a much lower concentration than a K-S test of distance moved and angular velocity while Nonafluoropentanamide was selected as it was not identified using either a K-S test of distance moved and angular velocity. Similarity between the results was determined by comparing fourth order polynomial curve fits with and a significance threshold of p < 0.05.”

Some editorial changes: On the referenced GitHub page from lines 384-385, the Autoencoder Model Design and the ten Jupyter Notebook file links appear to be broken; In line 29 should be “abnormal behavioral effects” instead of “abnormal behavioral”

Thank you for these comments. Line 29 has been corrected and the broken links have been repaired.

Based on the need to sufficiently address these points described above, I would recommend resubmitting the manuscript with major revision.

Reviewer #3: This manuscript submitted by Green and colleagues reports a method based on machine learning to improve identification of abnormal behaviour of zebrafish larvae. Considering that behaviour of zebrafish is a frequently used methods to evaluate (neuro)toxicity of chemicals and the large amount of chemicals not tested yet, any way to improve sensitivity or accuracy is of utmost importance.
The Ms is well organised and easy to read (this reviewer not being able to evaluate the computational part focused on the behavioural and fish parts) and globally acceptable for publication provided below comments are addressed.

We greatly appreciate the valuable input you provided. In order to improve the caliber of our manuscript, we have made the following changes based on your feedback:

- We added additional details regarding swimming behavior, morphological assessments, and experimental design.
- We updated reference formatting.

One point would deserve more in-depth discussion because some results are puzzling. Indeed, in some cases, a higher sensitivity can explain differences between statistical and autoencoder methods (Table 2), but in other ones (Multi-Walled Carbon Nanotube; 1-Methylphenanthrene, Dibenzo[e-l]pyrene, Aminohexafluoropropan-2-ol) differences are in the middle of the concentrations range. How the Authors can ensure they are not false positive?

This is an excellent question and addressed in Table 1 which shows that all of our autoencoder models have high specificity and high positive predictive values. In addition, we have shown that our results are reproducible (Fig. 4). As for the differences in the middle of the concentration range these indicate a lack of sensitivity in the traditional statistics that result that rely on summary metrics.

Given that the procedure appears to require high-level computational expertise, how can this help as many labs as possible benefit from this new approach?

Specific comments
L116 The Authors write "Zebrafish larvae show mature swimming patterns … at four to five days post-fertilization (dpf), which can be assessed using various locomotor behavioral assays…"
The sense of "mature swimming pattern" is not clear; it does not correspond to a specific situation. In addition 1) behaviour is very variable between 4 and 6 dpf and 2) response to chemicals varies significantly with often no response at 4 dpf while defect can be detected at 5 dpf. The sens of mature should be precisely defined (or the sentence rewritten) and 4 to 5 dpf should be distinguished in terms of maturity of responses.

Thank you for this comment. We have removed the term mature and added additional details regarding the swim patterns that have developed by 5 dpf: “Zebrafish larvae show swimming patterns essential for their survival following swim bladder development at four to five days post-fertilization (dpf), including exploration, foraging, and escape response which can be assessed using various locomotor behavioral assays (Hernandez et al., 2018; Tegelenbosch et al., 2012) while more advanced continuous swimming, schooling, and reproductive behavior is still developing.”

L256 "more recently" is not correct since most studies are older
L297 reference is made to Fig. 3 while ti should be Fig. 2.

Thank you. These errors have been corrected.

L318 type of 96 well plates should be indicated since round well plates have proven to produce high occurrence of swim bladder inflation defects. According to figures round well plates were used, so percentage of malformed larvae (see next comment) should be provided. Medium renewal or its absence should be clearly indicated.
L324 morphological defects scored at 24 and 120 hpf should be indicated

Thank you for this comment. As suggested, we have added additional details to the Methods - Mortality and morphology section: “At 24 hours post-fertilization (hpf), embryos were screened for mortality, developmental delay, and spontaneous movement(Truong et al., 2014). At 120 hpf, mortality, craniofacial abnormalities (eye, snout and jaw), body axis abnormalities, edema (yolk sac and pericardial edema), upright body abnormalities (swim bladder, somite and circulation), touch response brain abnormalities (brain, otic vesicle and pectoral fin), pigment, notochord, and trunk abnormalities (trunk and caudal fin) (Thunga et al., 2021; Truong et al., 2014; G. Zhang et al., 2016). The incidence of abnormality across all morphology endpoints were evaluated as binary outcomes. Any individuals identified with a physical abnormality were excluded from any behavioral analysis as these abnormalities might confound the results.”

L331-332 the recording protocol is not correctly described, 3 cycles of 3+3 phases are equivalent to 18 minutes and not 24. Perhaps because acclimation precedes the first cycle. This should be corrected, and for acclimation if any, was it in the light or in the dark?

Thank you for bringing with to our attention. You were correct there was a 6 minute acclimation period. This detail has been added to the Methods - Photomotor responses section:” The recorded videos were then tracked with Ethovision XT v.11 analysis software for 24 min across 3 cycles of 3 min light: 3 min dark with an initial 6 minute acclimation period.”
L335-336 so what is the purpose of this third cycle?

This is a good question. The third cycle provides a means of evaluating learning impairment and was not included with the scope of this analysis.

L544 ref format not correct

Thank you for this comment. The reference format has been changed to PLoS Computational Biology.

Even though Tanguay's laboratory has produced a large amount of data dealing with the behavior of zebrafish, a fairer representation of the literature should be presented!

**Have the authors made all data and (if applicable) computational code underlying the findings in their manuscript fully available?**
The [PLOS Data policy](https://journals.plos.org/ploscompbiol/s/materials-and-software-sharing) requires authors to make all data and code underlying the findings described in their manuscript fully available without restriction, with rare exception (please refer to the Data Availability Statement in the manuscript PDF file). The data and code should be provided as part of the manuscript or its supporting information, or deposited to a public repository. For example, in addition to summary statistics, the data points behind means, medians and variance measures should be available. If there are restrictions on publicly sharing data or code —e.g. participant privacy or use of data from a third party—those must be specified.

Reviewer #1: Yes

Reviewer #2: **No:** Two links on Github appear to be broken upon attempt to access: On the referenced GitHub page from lines 384-385, the Autoencoder Model Design and the ten Jupyter Notebook file links appear to be broken

It is stated that the data is available upon request instead of being uploaded to a public repository.

These links have been repaired and a subset of the data is available for easy access and review. The entire dataset is available upon request.

Reviewer #3: Yes

PLOS authors have the option to publish the peer review history of their article ([what does this mean?](https://journals.plos.org/ploscompbiol/s/editorial-and-peer-review-process#loc-peer-review-history)). If published, this will include your full peer review and any attached files.


If you choose “no”, your identity will remain anonymous but your review may still be made public.


**Do you want your identity to be public for this peer review?** For information about this choice, including consent withdrawal, please see our [Privacy Policy](https://www.plos.org/privacy-policy).

Reviewer #1: No

Reviewer #2: No

Reviewer #3: No

Figure Files:

While revising your submission, please upload your figure files to the Preflight Analysis and Conversion Engine (PACE) digital diagnostic tool, [https://pacev2.apexcovantage.com](https://pacev2.apexcovantage.com/). PACE helps ensure that figures meet PLOS requirements. To use PACE, you must first register as a user. Then, login and navigate to the UPLOAD tab, where you will find detailed instructions on how to use the tool. If you encounter any issues or have any questions when using PACE, please email us at [figures@plos.org](mailto:figures@plos.org).

Data Requirements:

Please note that, as a condition of publication, PLOS' data policy requires that you make available all data used to draw the conclusions outlined in your manuscript. Data must be deposited in an appropriate repository, included within the body of the manuscript, or uploaded as supporting information. This includes all numerical values that were used to generate graphs, histograms etc.. For an example in PLOS Biology see here: <http://www.plosbiology.org/article/info%3Adoi%2F10.1371%2Fjournal.pbio.1001908#s5>.

Reproducibility:

To enhance the reproducibility of your results, we recommend that you deposit your laboratory protocols in protocols.io, where a protocol can be assigned its own identifier (DOI) such that it can be cited independently in the future. Additionally, PLOS ONE offers an option to publish peer-reviewed clinical study protocols. Read more information on sharing protocols at <https://plos.org/protocols?utm_medium=editorial-email&utm_source=authorletters&utm_campaign=protocols>
