# Supplementary material for: Deep autoencoder-based behavioral pattern recognition outperforms standard statistical methods in high-dimensional zebrafish studies
Source: PLoS Comput Biol. 2024 Sep 10;20(9):e1012423. doi: 10.1371/journal.pcbi.1012423 (PMC11414989; doi:10.1371/journal.pcbi.1012423)
Supplement: Attachment — Submitted filename: Author response to Reviewers_July2024.docx [file pcbi.1012423.s006.docx]

Reviewer's Responses to Questions

**Comments to the Authors:
Please note here if the review is uploaded as an attachment.**

Reviewer #2: Thank you for including thorough responses to the majority of the issues that I addressed in my initial review. The few items remaining to address are included below:

Supplementary figures or table information should be included to display the directionality of the effects described in Supplementary Table 2 and Supplementary Table 3. As is, it is unknown whether the chemicals cause an increase or decrease in each of the distance traveled and angular velocity measures for each chemical. Please include figures showing this or information in a supplementary table showing the mean and standard deviation (or standard error) and number of samples for each chemical and control levels for both distance traveled and angular velocity.

Thank you for this excellent suggestion. Supplementary Tables 2 and 3 have been replaced with a new Supplementary Table 2 as an Excel spreadsheet with the requested data. While doing this we identified a couple errors and updated the manuscript accordingly (lines 166 – 171 and Table 2).

Please address the data availability issue described in the data availability section. I have included this here for reference: The full set of data and code are not provided as part of the manuscript or its supporting information or deposited to a public repository. Although part of the data are provided the manuscript states that you need to contact the authors to access the full dataset and code. This does not yet meet the PLOS Data policy description.

A full dataset has been made available with the code. The manuscript has been updated to reflect this change (lines 397 to 399).

Reviewer #3: Thanks for having addressed most of the comments raised. Could you elaborate a bit about the way other laboratories will be able to adopt this approach on the one hand and on the other hand the flexibility of the behavioral protocol (since there is no standard protocol).

This is a good point and has been addressed in lines 290 – 292. While each lab would need to train their own deep autoencoder to account for differences experimental design and strain. The resources required to do so are not extensive and, in most cases, historical data would likely be available.

Second point please provide information about lighting during acclimation.

Information added to line 347.
